# Beyond IgE—When Do IgE-Crosslinking and Effector Cell Activation Lead to Clinical Anaphylaxis?

**DOI:** 10.3389/fimmu.2017.00871

**Published:** 2017-08-10

**Authors:** Lars K. Poulsen, Bettina M. Jensen, Vanesa Esteban, Lene Heise Garvey

**Affiliations:** ^1^Allergy Clinic, Copenhagen University Hospital at Gentofte, Hellerup, Denmark; ^2^Immuno-Allergy Laboratory, Department of Immunology, IIS Fundación Jiménez Díaz, Madrid, Spain

**Keywords:** anaphylaxis, allergens, mast cells, mast cell activation, cofactors

## Abstract

Anaphylaxis in humans is inherently difficult to study due to the acuteness of symptoms and the lack of biomarkers serving as risk predictors. Most cases are related to IgE sensitizations to foods, insect venoms, and drugs with mastocytosis patients forming a smaller risk group. However, identifying the relatively small fraction of persons at risk has been exceedingly difficult. In this review, we propose to describe anaphylaxis in a broader context than defined by IgE sensitization alone. Exposure to a trigger, such as an allergen, may lead to anaphylaxis, but in particular, the internal dose sensed by the immune system needs to be established. Moreover, intrinsic patient factors as well as the specific circumstances of the exposure, i.e., the extrinsic factors, need to be thoroughly accounted for. More controversially, other triggers of anaphylaxis, such as increased sensitivity to or reduced catabolism of histamine (“histamine intolerance”) or mast cell activation syndrome also named mast cell activation disorder have been suggested, but still with very limited epidemiological evidence that a significant proportion of the observed reactions are caused by these alleged conditions. Thus, when all conditions are considered, it seems as if IgE-mediated reactions are responsible for the vast majority of anaphylactic conditions.

## Introduction

Anaphylaxis is a serious allergic reaction that is rapid in onset and may cause death (1). It typically involves one or more of the symptoms: an itchy rash, throat or tongue swelling, shortness of breath, vomiting, lightheadedness, and low blood pressure (BP) appearing in minutes to hours after a stimulus that is mostly believed to be of exogenous origin.

Epidemiological studies are scarce (1), but recent studies suggest that foods (two-thirds), insect venoms (20%), and drugs are among the most frequent triggers, with differences between age groups. A special population at risk seems to be patients with mastocytosis, where various triggers including mechanical stimuli and insect stings—even without the presence of allergic sensitizations to venom allergens—may precipitate severe reactions.

While becoming increasingly popular in quasi-scientific fora on the Internet, other triggers of anaphylaxis, such as increased sensitivity to or reduced catabolism of histamine (“histamine tolerance”) or mast cell activation syndrome (MCAS) also named mast cell activation disorder (MCAD) still have unclear definitions and limited epidemiological evidence exists that a significant proportion of anaphylaxis should be caused by these alleged conditions. Thus, when all conditions are considered, it seems as if IgE-mediated reactions are responsible for the vast majority of anaphylactic conditions.

When looking at the triggers of anaphylaxis, it is clear, however, that only a tiny fraction of patients with food, insect venom, or drug allergy will experience an anaphylactic reaction. Due to the severity and life-threatening nature of anaphylactic reactions, it is of utmost importance to identify the risk factors in these patients that may predict—or ultimately prevent—the occurrence of anaphylactic reactions. While some foods seem to be more allergenic than others, there is little prognostic value in both food sources and sensitization to specific allergens. A similar uncharacteristic pattern has emerged for insect venoms, and even more so for drugs where the diversity and complexity of drugs make it impossible to gather much experience except for large drug groups such as beta lactam antibiotics.

Next in line for the risk analysis comes quantitative factors, such as the IgE-titer—or more indirectly: skin test response—to certain allergens. Also, these parameters have, however, failed to be strongly predictive of anaphylaxis risk, and examples can be found where the IgE-titer in serum over time has fallen below the detection limit, while the patient has retained clinicial reactivity upon reexposure (2). While IgE sensitization is still the best biomarker for risk of anaphylaxis, we aim at identifying additional features of the anaphylaxis pathophysiology that may eventually provide tools for identifying the patients at high risk.

**Figure 1:**
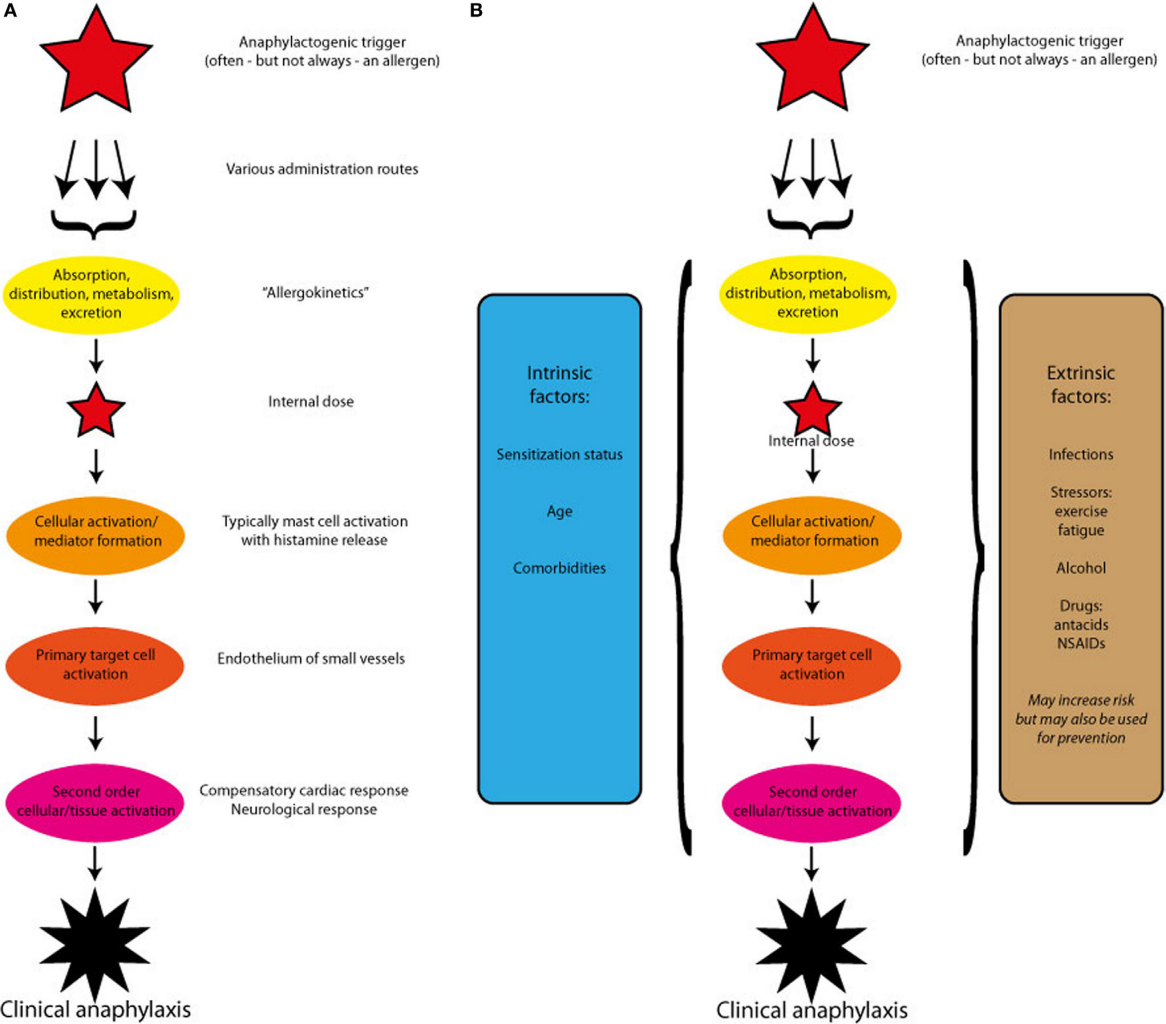
**(A)** A theoretical outline of the pathogenesis of anaphylaxis induced by a single trigger. After absorption and redistribution, the trigger reaches and activates the effectors cells. The ensuing mediator release induces the primary target cells to react, which may in turn activate compensatory or enhancing mechanisms. **(B)** A theoretical outline of the pathogenesis of anaphylaxis induced by a single trigger, but modified by host-specific intrinsic and extrinsic factors. The modifying factors may alter each of the steps: cellular activation, primary target cell activation, or secondary cellular or tissues activation.

All the above evidence suggests that individual factors to a great extent determine the severity of the allergic response and thereby also the risk of developing anaphylaxis, but knowledge about predictive factors is lacking. In this paper, we propose a theoretical framework for the pathogenesis of anaphylaxis, which by investigating a putative pathway of the mast cell activation, the primary target cells of the mast cell mediators, and of the neurological and other secondary mechanisms in the vasculature display a research paradigm that may help shedding some light on a disease, which by its acute form and unpredictable occurrence has so far eluded a more systematic study approach (Figure 1A).

## Type 1 Hypersensitivity

The hypersensitivity reaction classified by the British immunologists, Philip Gell and Robin Coombs, as the type 1 reaction (now also named *the IgE-mediated allergic reaction*, but notably IgE had still not been discovered and named in 1963, when Gell and Coombs first suggested their classification of this humoral form of hypersensitivity), still provides the basic theoretical framework for our discussion of anaphylaxis. Centrally in this theory is the involvement of effector cell-bound antibodies, which upon binding to an allergen will induce activation and release of anaphylactogenic mediators. Immunoglobulin E is in humans the only antibody class with a solid evidence of binding to and activating the effector cells, mast cells, and basophil granulocytes, *via* the tetrameric form of the high affinity IgE receptor, the FcεRI comprising one alpha, one beta, and two gamma units (as opposed to the trimeric form of the receptor, lacking the beta-chain, which may be found on other cell types not believed to take part in the acute allergic reaction). So-called homocytotrophic antibodies, i.e., antibodies binding to effector cells, of other isoforms such as IgG4 (3), IgD (4), and even isolated light chains (5) have been suggested, but not supported by a solid body of evidence. There is likely to be marked differences between man and rodents, and while the human isotype IgG4 has similarities to mouse, rat, and guinea pig IgG1, there is little evidence of functional effector cell receptors playing an important clinical role in the human system. Thus, it seems fair to conclude that most anaphylactogens are in fact allergens, as defined as an antigen to which an IgE-immune response is mounted.

As mentioned above, there is a large body of literature discussing the quality and quantity of IgE, in relation to anaphylaxis caused by foods, insect venoms, and drugs. Many such studies suggest that not all allergens may necessarily be anaphylactogens, but since our aim is to go beyond this discussion, we will not dwell further on this aspect, but refer the reader to the vast amount of literature most recently reviewed in Ref. (6).

## Allergokinetics and Internal Dose

Much less studied are the pathways before and after the sensitized effector cell meets the allergen (Figure 1A). We have used the term *allergokinetics* (derived from the terms and concepts of pharmacokinetics and toxicokinetics) to describe the mechanisms by which an allergen source such as a food, an insect venom, or a drug is taken up by the body, how the allergenic (=IgE-binding) molecules are solubilized, absorbed over biological membranes, and eventually distributed in the organism where it is able to meet and activate effector cells, causing mediator release. Even less studied are the subsequent events where the allergen is potentially metabolized and excreted by the organism.

We propose to use the concept of *internal dose*, to describe the quantity of the anaphylactic trigger (most often an allergen) that becomes available to the systemic circulation, from where it is believed to activate the vascular system.

Old studies using the method of passively sensitizing healthy persons with a hyperimmune serum from a sensitized person, followed by ingestion of the culprit allergenic food, has demonstrated a much faster uptake than would be expected by normal gastrointestinal physiology (7). This was confirmed by studies using sensitive biological tests (8, 9) for detection of allergen in plasma of non-allergic persons ingesting foods to which hyperimmune sera could be used for detection. Finally, an elegant revival of the old Prausnitz–Küstner method has been used after a rigorous ascertainment of the safety of the sensitizing serum. Here, an inverse relationship between oral dose and onset of skin reaction was demonstrated (10). The overall conclusions from these studies suggest that less than one part per million of food protein (and thus almost out of reach with the present status of analytical allergen detection) is absorbed systemically. However, so far, it has not been possible to study whether similar mechanisms of limited intact food allergen absorption is taking place in allergic patients, or whether they might have qualitatively different mechanisms operating.

Some foods are known to potentially induce severe anaphylactic reactions, such as egg and milk in early childhood, and peanut, tree nuts, fish, and shellfish persistently throughout life. It is well-accepted that a “dangerous” food contains allergens that are sufficiently resistant to gastrointestinal proteolysis allowing them to cross the gut epithelial barrier and cause systemic reactions. Legume and tree nut storage proteins such as peanut Ara h 2 and lipid transfer proteins such as peach Pru p 3 are probably the best characterized molecules in this category. They are both highly resistant to proteolysis and have been identified as risk factors for severe symptoms. Nevertheless, a very significant number of patients sensitized to such stable allergens only experience mild symptoms, usually limited to the oral cavity. This means that, besides protease-resistance, other factors determine the clinical outcome of exposure to potentially severe (stable) allergens.

The relationship between the *internal dose* reaching the immune system, and the actual dose administered *via* ingestion, inhalation, skin absorption, or *via* parenteral routes is not only determined by absorption. In particular, many food allergens are digested in the stomach but may also react or become absorbed intact in the buccal mucosa (8). The clinical studies of threshold values to allergenic challenges suggest that there are large interindividual (of 3–5 decades) differences between patients’ threshold dosages when challenged with food allergens. Although insufficient data are available, it seems most likely that each individual has his/her own threshold value, and the more the given dose exceeds this threshold, the higher likelihood of an anaphylactic reaction.

## Mediator Release and Primary Target Cell Activation

Following mediator cell activation, different mediators, the most prominent being histamine, but likely also involving tryptase, prostaglandins, the sulphido-leukotrienes, LTC4, LTD4, and LTE4; platelet aggregating factor and other lipid-derived mediators are released to the surrounding tissue to act locally and systemically. Both primary triggers such as allergens and mediators released by these may be systemically distributed, but it is rarely clear which of the two distribution mechanisms that are most important for the systemic nature of anaphylaxis.

## Secondary Effects: Localized or Systemic Response?

Anaphylaxis can affect multiple organ systems and results in a broad range of symptoms from the skin and mucous membranes, upper and lower respiratory tract, gastrointestinal tract, and cardiovascular and nervous systems. Cutaneous symptoms are present in more than 80% of episodes and are often transient including erythema, pruritus, rash, and urticaria/angioedema (11, 12). During skin and mucosal symptoms, the dermal microvasculature is highly unbalanced and important extravasations of fluid occurs. However, the vascular system extends well beyond of the microvasculature. The majority of severe alterations described in human anaphylaxis involve the vascular system and hypotension is one of three important criteria for diagnosing anaphylaxis, with resulting hypoxia being a key feature contributing to the severity of the reaction (13, 14). Overall hemodynamic defects are fundamental for the sudden fall in the BP and may directly cause some of the neurologic symptoms as dizziness, fainting, and seizures associated to severe anaphylaxis. Moreover, involvement of the gastrointestinal system (abdominal pain, cramping, nausea, vomiting, incontinence, and diarrhea) has been strongly correlated with hypotension and hypoxia too. Low BP during anaphylaxis might result in decreased myocardial perfusion, which in turn causes arrhythmias and cardiovascular collapse. The respiratory system is commonly affected giving rise to symptoms such as dyspnea, wheezing, stridor, deep cough, upper airway obstruction, asphyxia, and respiratory arrest (15, 16).

The extravasation of fluid leads to decreased venous return, which in turn causes insufficient filling of the heart, reduced cardiac output, and ultimately cardiac arrest. Respiratory obstruction/arrest, cardiovascular collapse, or a combination of these might be fatal. Moreover, there is increasing evidence of the human heart as a target of cardiac anaphylaxis involving human heart mast cells (17), but this area deserves further studies.

## Patient-Associated Intrinsic Factors

Clearly, allergen dose and the patients IgE titer are not enough to predict the severity of an anaphylactic reaction, and other contributing factors termed cofactors may be described as intrinsic or extrinsic (Figure 1B).

Patients with, e.g., severe food allergy often report an almost immediate tingling sensation in the mouth, already upon exposure to tiny quantities of the offending food. An anaphylactic reaction can occur within a few minutes, so symptoms are initiated long before the food allergen has had the chance to pass the stomach into the intestinal tract and interact with the relevant epithelial mast cells. This suggests that the process of anaphylaxis already starts when the allergen is exposed to the oral (or esophageal) mucosal/epithelial barrier. The kinetics of uptake of food proteins over the oral mucosal barrier is a poorly understood process that may differ between patients with mild and severe food allergy. Anaphylactic patients may have a more permeable mucosal/epithelial barrier in the oral cavity but possibly also in the intestinal tract. In addition to differences in the physical barrier function, the innate immune function of epithelial cells may also differ, and the number of subepithelial tissue mast cells and/or their sensitivity to allergen may distinguish between patients with mild and severe reactions. Also, differences in the phenotype and thus responsiveness of mast cells (18) and basophils (19) may play a role.

In addition, much more crude physiological parameters, such as age, hormonal status, and comorbidities may determine the reactivity of the organism being exposed to an anaphylactic trigger, and it is important to emphasize that the intrinsic, as well as the extrinsic factors described below, may interact differently with each of the steps in the anaphylactic pathway.

## Extrinsic Factors: Food, Lifestyle, Environment, and Infections

Thresholds for allergic reactions to food also have considerable intraindividual variability. It is nevertheless assumed that all patients can develop severe reactions, given the right combination of factors and events and if the dose is high enough. Apart from the dose of a food, there is also the way it is consumed, i.e., pure unprocessed, processed, and/or as part of a composite food. The matrix in which a food allergen is presented to mucosal surfaces, both with respect to its composition and its way of processing, is an extrinsic factor that has significant impact on release and uptake of food allergens (Figure 1B).

Other extrinsic factors that may influence allergen uptake are exercise shortly after food consumption (exercise-induced anaphylaxis), alcohol-use, and the use of non-steroidal antiinflammatory drugs and antacids (20, 21). Exercise-induced anaphylaxis is most commonly but not exclusively associated with wheat allergy. All these extrinsic factors are thought to increase gut permeability or increase allergen solubility, thereby lowering the threshold for severe reactions. Quantitative data are, however, not available. Some of these extrinsic factors may very well synergize into the perfect storm of a “party challenge” (dinner including potential allergens, alcohol, dance, preventive aspirin, antacid), of which the quantitative impact is of course even more complex. Stress and sleep deprivation are also on the list of extrinsic factors that increase the risk for severe reactions. Last but not least, infections such as common cold (rhinovirus) and flu (influenza virus) have been implicated to increases hyperresponsiveness and may thus lower thresholds or increase the severity of a reaction. As of yet, the effects attributed to most of these extrinsic factors have not yet been confirmed by clinical, prospective studies.

## Triggers of the Anaphylactic Reaction

**Figure 2:**
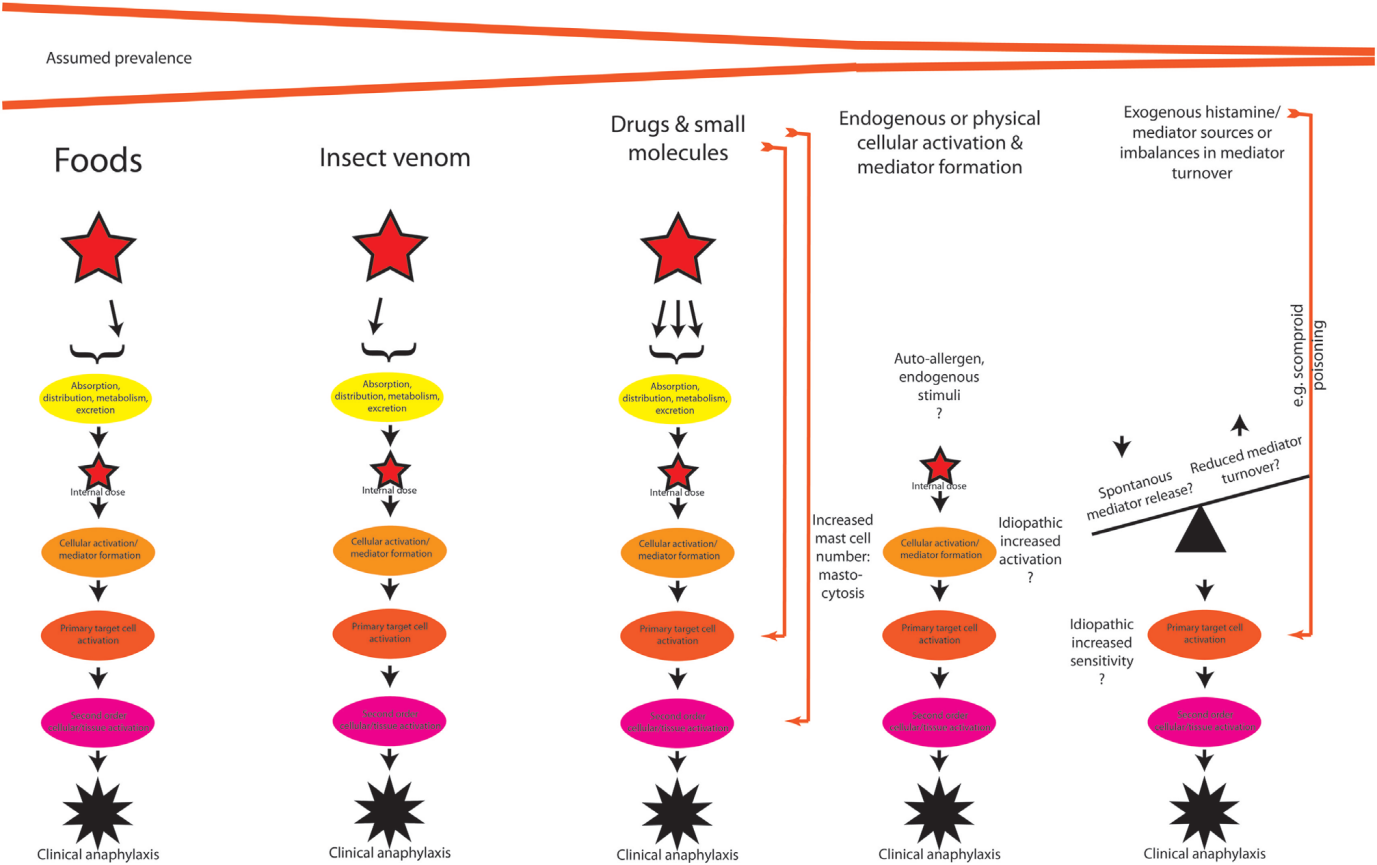
A theoretical outline of the pathogenesis of anaphylaxis induced by different triggers, with potentially different pathways. See text for discussion.

After having discussed the potential host-modifying (intrinsic and extrinsic) factors (Figure 1B), we can move back to the actual triggers (Figure 2) of the reaction. Clearly, not all clinical cases of anaphylaxis may be fully explained and diagnosed, but as previously cited from the literature, food seems to be most important trigger, followed by insect venom stings and drugs, the latter also including allergic side effects of allergen-specific immunotherapy, and systemic reactions caused by diagnostic (iatrogenic) allergen challenges.

### Food

By definition, the oral route is the only relevant exposure to foods, even though food proteins are known to elicit (and perhaps sensitize) *via* inhalation or *via* dermal exposure. This does not preclude, however, that the primary sensitization to a food allergen has taken place *via* another administration route.

### Insect Venoms

Insect stings often occur in body areas with thin skin or on mucous membranes that allow a rapid and relatively unhindered systemic absorption of the individual venom components. By nature, these are toxic in their own right, and even if the patients is not allergic, large local reactions may be seen.

### Drugs

Anaphylactic reactions to drugs are divided into allergic reactions (including IgE-mediated reactions) and non-allergic reactions, which may have several other mechanisms, such as activation *via* the complement system or direct actions of a drug on receptors of the target cell, e.g., opioid drugs activating mast cells directly, or more recently, the suggestion that some drugs may directly activate the mast cell *via* the MRGPRX2 surface receptor (22).

In practice, it is important not to limit the scope to the active component in prescription drug. Excipients and additives such as, e.g., methylcelluloses or polyethylene glycols (23) may also have allergenic potential. In addition, chemicals used for disinfection or sterilization such as chlorhexidine and ethylene oxide may be important to consider. Also, many drugs have potent effects in their own right, and the differentiation between pharmacological effects, side effects, and allergic symptoms is important for reaching the correct diagnosis (24).

Within the realm of perioperative anaphylaxis and postsurgical recovery, the administration of blood products may also give rise to anaphylactic reactions, and here, two immune systems, the host’s and donor’s, may be on collision course with other mechanisms than IgE being involved. Such mechanisms includes an immune-complex-mediated (with possible complement-system involvement also including the contact system) reaction between anti-IgA auto-antibodies of IgA-deficient donors and IgA of the recipient (25).

### Endogenous Cellular Activation: Mastocytosis and Other Mast Cell-Related Disorders

Mastocytosis is a well-known cause of anaphylaxis (26), which can often be diagnosed *via* the signature KIT D816V mutation (27). Additionally, condition(s) described as MCAS or MCAD have been suggested, but it has been difficult to establish firm criteria for this or these conditions. One important step forward was the suggestion to first eliminate primary MCADs such as mastocytosis and secondary activation disorders such as allergic reactions and other conditions caused by receptor-mediated mast cell activation before considering MCAS. Further, strict criteria have been proposed including (a) symptoms from two or more organs; (b) response to anti-mediator therapy; and (c) evidence of increased mediator turnover (28). It seems fair to state that the clinical significance of such reactions are still controversial and very limited epidemiological evidence exists where the above-described criteria have rigorously been applied. Thus, when all conditions are considered, it seems as if IgE-mediated reactions followed by mastocytosis-related anaphylaxis are responsible for the vast majority of anaphylactic conditions.

### Exogenous Mediator Intake and/or Reduced Catabolism, i.e., Histamine Intolerance

Scombroid poisoning—an allergy-like intoxication after ingestion of fish or other foods with high histamine content due to microbial degradation—can be considered as an overdose of histamine. Intake of more than 50 mg histamine/100 g food is considered toxic, but intake of histamine 20 mg/100 g food has in some cases elicited symptoms. None of the symptoms are pathognomonic to histamine poisoning, and the only reliable way to diagnose this condition is to measure histamine content in the food (29, 30).

It is likely that sensitive individuals exists, who may react to lower dosages of histamine, either because of an increased sensitivity at the receptor level or because of a reduced catabolism of histamine, which is mainly degraded by the enzyme, diamine oxidase [see p. 89 in Ref. (31)]. In principle, similar syndromes may exists for other mediators. A considerable literature is available, but the diagnosis, *histamine intolerance*, is controversial (32) and not always easily differentiated from extrinsic or intrinsic factors described above.

## Conclusion

Experimental evidence, both clinically and at laboratory level, is needed to facilitate better understanding of the mechanisms by which severe reactions occur, but also to be able to quantify their impact on threshold doses. Severe, potentially life-threatening anaphylactic reactions have great impact on the quality of life of patients. They are the basis of the anxiety and fear common to these patients and their relatives. An important knowledge gap relevant to evidence-based risk management of anaphylaxis is our poor ability to predict whether, and if so when, patients will develop such severe systemic, potentially life-threatening reactions. Here biomarkers are essential, and biomarkers that are bedded in mechanisms of disease are more powerful than markers identified simply by association (33). On the other hand, the difficulties in performing clinical research on patients with acute but fast remitting symptoms may also have inspired the proposal of a number of controversial disease mechanisms.

## Author Contributions

LP made the original outline after common discussions. BJ, VE, and LG read and commented the manuscript, which was finalized by LP.

## Conflict of Interest Statement

The authors declare that the research was conducted in the absence of any commercial or financial relationships that could be construed as a potential conflict of interest.
